# Outcomes of cochlear implantation in 75 patients with auditory neuropathy

**DOI:** 10.3389/fnins.2023.1281884

**Published:** 2023-11-13

**Authors:** Jie Wu, Jiyue Chen, Zhiwei Ding, Jialin Fan, Qiuquan Wang, Pu Dai, Dongyi Han

**Affiliations:** Key Lab of Hearing Impairment Science of Ministry of Education, Key Lab of Hearing Impairment Prevention and Treatment of Beijing, National Clinical Research Center for Otolaryngologic Diseases, College of Otolaryngology Head and Neck Surgery, Chinese PLA General Hospital, Chinese PLA Medical School, Beijing, China

**Keywords:** auditory neuropathy, cochlear implantation, impact factor, cochlear nerve, genetic testing

## Abstract

**Background:**

Cochlear implantation (CI) outcomes in patients with auditory neuropathy (AN) are variable, which hampers patients’ decisions on CI.

**Objective:**

This study aims to assess the outcomes of CI in individuals diagnosed with AN and to examine the various factors that may influence the effectiveness of this intervention.

**Methods:**

A total of 75 patients diagnosed with AN were included in the study. The hearing threshold, the score of categories of auditory performance (CAP), speech intelligibility rating (SIR), and speech audiometry were tested. Genetic testing was conducted by medical exome sequencing in 46 patients.

**Results:**

After CI, the average aided hearing threshold for patients with prelingual and post-lingual onset was 38.25 ± 6.63 dB and 32.58 ± 9.26 dB, respectively; CAP score improved to 5.52 ± 1.64 (*p* < 0.001) and 6.00 ± 0.96 (*p* < 0.001), respectively; SIR score increased to 3.57 ± 1.22 (*p* < 0.001) and 4.15 ± 0.95 (*p* < 0.001), respectively. Maximum speech recognition ranged from 58 to 93% for prelingual onset patients and 43 to 98% for those with post-lingual onset. Speech outcomes of CI in cases with cochlear nerve (CN) deficiency were significantly poorer (*p* = 0.008). Molecular etiologies, including *TWIST1*, *ACTG1*, m.*A7445G*, and a copy-number variant (CNV) carrying ACTB, were related to AN here.

**Conclusion:**

CI is a viable therapy option for patients with AN; CN deficiency might impact outcomes of CI.

## 1. Introduction

Auditory neuropathy (AN) or auditory neuropathy spectrum disorder (ANSD) is a specific type of sensorineural hearing loss characterized by the presence of otoacoustic emissions (OAE) and/or cochlear microphonics with the absent or abnormal auditory brainstem responses (ABR) (Moser and Starr, 2016). Nearly 9.85% of 1,025 children with sensorineural hearing loss were identified to have ANSD (Almishaal et al., 2022).

In patients with AN, the degree of hearing loss (HL) varies from mild to profound, and it can occur prelingually or post-lingually. In contrast, the hearing may be affected bilaterally or unilaterally. AN may occur simply or as part of some syndromes. Simultaneously, patients of AN may also have inner-ear malformations. Risk factors, including hyperbilirubinemia, infection, premature, ototoxic drug exposure, cochlear nerve (CN) deficiency, and congenital brain anomalies (Rajput et al., 2019; Natale et al., 2020; Liddle et al., 2022), are highly related to AN.

Many genes have been found as the molecular causes of AN (Moser and Starr, 2016; Shearer and Hansen, 2019), with hereditary patterns involving autosomal recessive, autosomal dominant, X-linked, and mitochondrial. More novel genes associated with AN are expected to be discovered, which would promote our insights into the pathogenesis of AN and guide the treatment and prevention of this disorder. Considering the treatment, cochlear implantation (CI) can benefit patients with AN (Sarankumar et al., 2018; Alzhrani et al., 2019). Studies have demonstrated that factors including the age of implantation, duration of cochlear, and genetic lesion sites could impact outcomes of CI (Daneshi et al., 2018; Shearer and Hansen, 2019). Speech performances in patients with AN receiving CI were variable (Harrison et al., 2015; Chaudhry et al., 2020), which traps the patients’ decision on CI.

This study enrolled 75 unrelated patients with AN who received cochlear implantation. Outcomes of CI in patients were evaluated through auditory assessment, speech audiometry, and questionnaires, including categories of auditory performance (CAP) and speech intelligibility rate (SIR). Meanwhile, for 46 patients, medical exome sequencing was performed for genetic testing. The molecular etiology of this specific patient subgroup was thoroughly elucidated. Furthermore, examining candidate factors, including hereditary influences that may affect the IC outcomes of individuals with AN, was also assessed to provide more reliable information for the decision of the rehabilitation approach for AN.

## 2. Materials and methods

### 2.1. Subjects

In this study, 84 patients diagnosed as AN and received CI in Chinese PLA General Hospital from 2010–08 to 2020–11 were enrolled. Exclusion criteria were: (1) having a tumor (e.g., acoustic neuroma), (2) severe cognitive abnormality hampering speech development, (3) otitis media, and (4) loss to follow-up. Finally, 75 subjects were included. The Ethics Committee of Chinese PLA General Hospital approved this study. It was conducted as per the Declaration of Helsinki. Written informed consent was obtained from the participants or the parents of the minors.

### 2.2. Audiology and speech recognition evaluation

Auditory brainstem responses (ABR), distortion-product otoacoustic emissions (DPOAE), cochlear microphonics, and tympanometry were conducted to diagnose AN. Pure tone audiometry (PTA) was used for auditory threshold determination in subjects >5 years old who could complete this testing; behavior audiometry was performed in subjects ≤5 years old, including strength vision audiometer (subjects >1 and ≤ 2.5 years old) and play audiometer (subjects >2.5 and ≤ 5 years old). ABR and auditory steady-state response (ASSR) were measured for patients who could not undergo the aforementioned auditory tests. The degree of hearing loss was determined by the average threshold at frequencies of 0.5, 1, 2, and 4 kHz or response thresholds in ABR. The level of HL was graded as mild (26–40 dB), moderate (41–55 dB), moderately severe (56–70 dB), severe (71–90 dB), and profound (>90 dB). To evaluate the hearing sensitivity pre-operation and post-operation, categories of auditory performance (CAP) were also used.

Hierarchic speech audiometry was estimated based on patients’ age and their capability of hearing and speech. The Mandarin Early Speech Perception (MESP) test was performed for patients aged 2 to 5 years to evaluate the children’s recognition of words. The Mandarin Pediatric Speech Intelligibility (MPSI) test was chosen for patients aged 3 to 6 years to assess the children’s perception of sentences (Zheng et al., 2009a,b; Ji et al., 2015). Speech audiometry was performed to test Mandarin monosyllables, disyllables, and sentences in quiet for subjects who could complete this test (Ji et al., 2014). Additionally, speech intelligibility rating (SIR) was used to evaluate the communication ability before and after IC at least six months post-operation (Ji et al., 2015).

### 2.3. Imaging tests

In order to assess the condition of the inner ear and auditory nerve, a comprehensive diagnostic approach was employed, which included high-resolution computed tomography (CT) scanning of the temporal bone, magnetic resonance imaging (MRI) of the brain, and magnetic resonance hydrography (MRH) of the inner ear.

### 2.4. Genetic testing

The genetic cause of patients was identified through medical exome sequencing (Trio exome sequencing was performed for probands whose parents’ DNA samples were available). The peripheral blood was obtained, and DNA was extracted according to the standard protocol. DNA sequencing (targeting 52.9 Mbp of the genome, covering the exons of 19,608 genes), bioinformatics analysis, and variant interpretation were performed following an earlier described protocol (Wu et al., 2022). Copy number variation was detected as described earlier (Baux et al., 2017), and the method of in-run correction and the Z-test analysis was applied in CNV calling. Candidate variants were confirmed by Sanger sequencing, while CNV was confirmed by real-time quantitative PCR. Variants were classified based on the American College of Medical Genetics and Genomics guidelines. Novel variants identified here were submitted to the ClinVar database with the accession number from SCV002568090 to SCV002568102.

### 2.5. Statistical analysis

Mann–Whitney *U* test in SPSS Statistics 25 was applied for significance analysis. The statistical significance was defined as *p* < 0.05.

## 3. Results

### 3.1. Information of subjects

Of the 75 patients enrolled here, 61 had prelingual HL with the onset or awareness age ≤ 3 years old, and the remaining 14 subjects had post-lingual HL (>3 years old). Among this subgroup, the onset or awareness age of HL in 13 patients ranged from 3 to 16 years of age. Unilateral AN was identified in three cases, with sensorineural HL determined in the contralateral ear. The average implantation age was 2.67 ± 2.17 years for subjects with prelingual HL and 20.29 ± 8.44 years for patients with post-lingual HL. Bilateral implantation was conducted in eight cases with the same type of device (Table 1).

**Table 1:** Clinical details of 75 patients with AN enrolled in this study.

Feature	Number	Percentage
Male	39	52.00%
Female	36	48.00%
Onset		
Prelingual	61	81.33%
Post-lingual	14	18.67%
Onset/awareness age	Average ± SD (years)	
Prelingual		1.12 ± 0.69
Post-lingual		10.82 ± 5.16
Implantation age	Average ± SD (years)	
Prelingual		2.67 ± 2.17
Post-lingual		20.29 ± 8.44
Affected ear
Bilateral	72	96.00%
Unilateral	3	4.00%
Implanted ear		
Unilateral (L)	25	33.33%
Unilateral (R)	42	56.00%
Bilateral	8	10.67%
Type of cochlear		
Concerto F28	16	21.33%
Sonata F28	15	20.00%
CI24RCA	9	12.00%
CI24RE	9	12.00%
CI512	9	12.00%
HiRes 90 k	7	9.33%
Nurotron	5	6.67%
Pulsar+	3	4.00%
CI24K	1	1.33%
CI522	1	1.33%

### 3.2. Risk factors and comorbidities

After a careful review of the disease history of the patients, risk factors related to AN were identified in 14 patients, including neonatal hyperbilirubinemia (*n* = 3), neonatal hyperbilirubinemia combined with Kawasaki syndrome (*n* = 1), neonatal hyperbilirubinemia combined with favism (*n* = 1), neonatal RH hemolysis combined with acute bilirubin encephalopathy (*n* = 1), meningitis (*n* = 1), neonatal septicemia combined with intrauterine distress and mixed acidosis (*n* = 1), hypoxic–ischemic encephalopathy (*n* = 1), premature (*n* = 1), trauma (*n* = 1), expose to ototoxic medicine (*n* = 1), hydrocephalus (*n* = 1), and development delay (*n* = 1) (Supplementary Table S1).

Based on imaging results, 10 patients had bilateral cochlear nerve (CN) deficiency, including hypoplastic CN and absent CN; two cases had a bilateral cochlear malformation, and another case had enlarged vestibular aqueduct (EVA) (Supplementary Table S1; Sennaroğlu and Bajin, 2017).

Other relevant comorbidities included epilepsy (*n* = 1) and unilateral renal agenesis (*n* = 1).

### 3.3. Diagnosis of auditory neuropathy

Tympanograms of all 147 ears, including 74 left and 73 right ears (three cases with unilateral AN), were normal as A type. For the ABR test, 140 ears had no response to stimulants (69 left ears, 71 right ears), while the response threshold of the remaining seven ears ranged from 75 dB to 100 dB. Of 74 left ears, 9, 9, 9, 26, 33, 28, 40, 37, 39, 38 ears passed DPOAE at frequencies 0.125, 0.25, 0.5, 1, 2, 1.5, 3, 4, 6, 8 kHz, respectively, while 7, 7, 8, 26, 34, 33, 39, 38, 38, 35 of 73 right ears passed DPOAE at frequencies as mentioned above, respectively. In 58 left ears and 55 right ears, cochlear microphonics waveforms were detected.

### 3.4. Auditory and speech assessments before cochlear implantation

**Figure 1:**
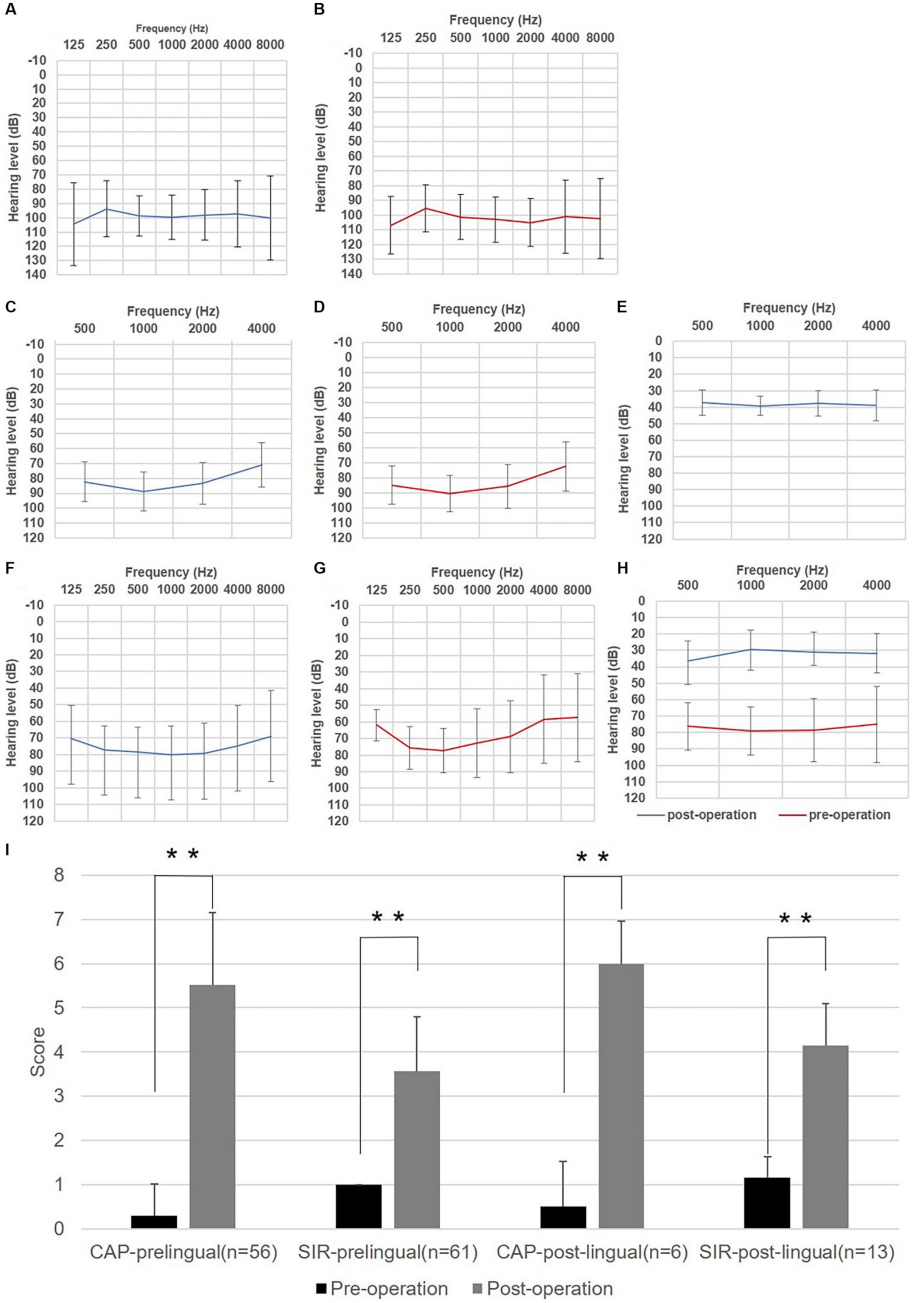
Auditory and speech outcomes before and after cochlear implantation (CI) in enrolled auditory neuropathy patients. **(A)** Hearing thresholds of left ears for subjects with prelingual onset determined by behavior audiometry (*n* = 20). **(B)** Hearing thresholds of right ears for subjects with prelingual onset determined by behavior audiometry. **(C)** Hearing thresholds of left ears for subjects with prelingual onset determined by the auditory steady-state response (ASSR) (*n* = 56). **(D)** Hearing thresholds of right ears for subjects with prelingual onset determined by ASSR. **(E)** Average aided hearing threshold after CI for subjects with prelingual onset determined by behavior audiometry (*n* = 30). **(F)** Hearing thresholds of left ears for subjects with post-lingual onset determined by pure tone audiometry (*n* = 11). **(G)** Hearing threshold of right ears for subjects with post-lingual onset determined by Pure tone audiometry (PTA). **(H)** Average aided hearing threshold after CI for subjects with post-lingual onset determined by PTA (*n* = 11). **(I)** Categories of auditory performance (CAP) and speech intelligibility rating (SIR) scores of enrolled subjects before and after CI. Whitney U test was applied for significance analysis, and statistical significance was defined as ^**^p < 0.01.

Of the patients with prelingual HL (61 subjects), behavior audiometry was completed in 20 cases, with the average hearing threshold being 98.38 ± 16.28 dB for left ears and 102.56 ± 17.14 dB for right ears (Figures 1A,B). ASSR results of this patient subgroup (*n* = 56) are shown in Figures 1C,D, with the average hearing threshold being 81.48 ± 15.23 dB for left ears and 83.34 ± 15.40 dB for right ears.

For the remaining 14 cases with post-lingual onset, the average hearing threshold through PTA was 79.08 ± 15.11 dB for left ears and 74.83 ± 22.58 dB for right ears (Figures 1F,G). At the same time, maximum speech recognition scores ranged from 0 to 36% (*n* = 5) for this subgroup by speech audiometer, indicating profound speech disabilities.

### 3.5. Auditory and speech performances after cochlear implantation

After operation, behavior audiometry was conducted in thirty cases with prelingual HL, and the average aided hearing threshold was 38.25 ± 6.63 dB, with the average hearing threshold at a frequency of 0.5, 1, 2, 4 kHz being 37.17 ± 7.62, 39.17 ± 5.88, 37.67 ± 7.85, and 39.00 ± 9.32 dB, respectively (Figure 1E), implying that subjects obtained good practical hearing due to CI. After implantation, the CAP score of the patients with prelingual HL (*n* = 56) improved from 0.29 ± 0.73 to 5.52 ± 1.64 (*p* < 0.001), and the SIR score (*n* = 61) increased from 1.00 ± 0.00 (before implantation, connected speech was unintelligible for all patients of this subgroup, with the primary mode of communication being manual) to 3.57 ± 1.22 (*p* < 0.001) (Figure 1I). Five patients of this subgroup completed speech audiometry post-operation, and four patients’ max speech recognition scores were > 60% (Table 2).

**Table 2:** Speech recognition rate of subjects after cochlear implantation.

Subject	Mandarin early speech perception test (MESP)				Mandarin pediatric speech intelligibility (MPSI)		Speech audiometry		
	Vowel	Consonant	Disyllable	Tone	The sentence in a quiet field	The sentence in the noise field	Monosyllable	Disyllable	Sentence
Prelingual									
1	40%	40%	58%	56%					
13							84%	77%	80%
20							72%	86%	93%
34							68%	61%	26%
75					92%	83% (SNR = +10 dB)			
Post-lingual									
8							52%	52%	38%
22	36%	36%	36%	56%			43%	36%	30%
43							33%	27%	96%
45							64%	55%	90%
51							82%	88%	98%

SNR, signal-to-noise ratio.

Among post-lingual hearing loss patients (*n* = 11), the average hearing threshold after CI through pure tone audiometry improved from 78.33 ± 17.34 dB to 32.58 ± 9.26 dB (*p* < 0.001) (Figure 1H). CAP score (*n* = 6) was enhanced from 0.50 ± 1.03 to 6.00 ± 0.96 (*p* < 0.001), and SIR score (*n* = 13) increased from 1.15 ± 0.48 to 4.15 ± 0.95 (*p* < 0.001) (Figure 1I). In this subgroup, the maximum speech recognition scores ranged from 52 to 98% for five subjects who underwent speech audiometry after CI (Table 2).

### 3.6. Outcomes of cochlear implantation in patients caused by genetic etiologies

In this study, 46 patients (39 with the prelingual onset and seven with post-lingual onset) participated in genetic testing using high throughput sequencing. When only pathogenic or likely pathogenic variants were considered, molecular etiologies were identified in 25 patients (20 patients of prelingual onset and five cases of post-lingual onset), with two leading responsive genes being *OTOF* in 13 prelingual onset patients and *AIFM1* in three cases of post-lingual hearing loss. Genetic testing results are presented in Table 3.

**Table 3:** The molecular causes of 25 diagnosed subjects.

Subject	Gender	Onset age	Gene	Variant	Functional consequence	Zygosity	Inheritance pattern	Reference	ACMG classification
11	F	0	*TWIST1*	NM_000474.3:c.309C>A:p.Tyr103Ter	Nonsense	Not determined	AD	rs104894054	P
20	M	1	*OTOF*	NM_194248.2:c.4493T>A:p.Val1498Glu	Missense	Het	AR	Novel	LP
			*OTOF*	NM_194248.2:c.5782C>T:p.Arg1928Cys	Missense	Het	AR	rs898393464	LP
21	F	2	*OTOF*	NM_194248.2:c.5098G>C:p.Glu1700Gln	Missense	Het	AR	rs199766465	LP
			*OTOF*	NM_194248.2:c.2407-2delA	Splice&deletion	Het	AR	Novel	P
30	F	0	*TWNK*	NM_021830.5:c.1172G>A:p.Arg391His	Missense	Het	AR	rs556445621	LP
			*TWNK*	NM_021830.5:c.1217G>A:p.Arg406Gln	Missense	Het	AR	rs756073260	LP
31	F	0.83	*OTOF*	NM_194248.2:c.5570G>A:p.Gly1857Asp	Missense	Het	AR	PMID: 35982127	LP
			*OTOF*	NM_194248.2:c.5212_5214delATC:p.I1e1738del	Deletion	Het	AR	PMID: 35982127	LP
34	M	0	*OTOF*	NM_194248.2:c.3399C>A:p.Tyr1133Ter	Nonsense	Het	AR	rs1665126718	P
			*OTOF*	NM_194248.2:c.5833del:p.Ile1945Serfs*4	Frameshift	Het	AR	Novel	P
37	F	0	*OTOF*	NM_194248.2:c.3674C>G:p.Ser1225Cys	Missense	Het	AR	Novel	LP
			*OTOF*	NM_194248.2:c.3592dup:p.Leu1198ProfsTer94	Frameshift	Het	AR	Novel	P
39	F	0.5	*MT-CO1*	m.A7445G				rs199474818	P
40	F	0.5	*OTOF*	NM_194248.2:c.5566C>T:p.Arg1856Trp	Missense	Het	AR	rs368155547	LP
			*OTOF*	NM_194248.2:c.764A>C:p.Gln255Pro	Missense&splice	Het	AR	Novel	LP
47	M	1	*OTOF*	NM_194248.2:c.4030C>T:p.Arg1344Ter	Nonsense	Het	AR	rs1060499805	P
			*OTOF*	NM_194248.2:c.1432T>C:p.Trp478Arg	Missense	Het	AR	Novel	LP
55	F	0	*OTOF*	NM_194248.2:c.4110_4120dup:p.Lys1374ArgfsTer152	Frameshift	Het	AR	Novel	P
			*OTOF*	NM_194248.2:c.2215-1G>C	Splice	Het	AR	Novel	P
56	F	1		chr7:4721914-5800744del		Het (*de novo*)	AD	PMID: 27633570	P
61	M	2	*TIMM8A*	NM_004085.3:c.133-2A>G	Splice	Hemi	XLR	rs1926076610	P
62	F	1	*OTOF*	NM_194248.2:c.5815C>T:p.Arg1939Trp	Missense&splice	Hom	AR	rs368790049	P
64	F	2	*OTOF*	NM_194248.2:c.5291 + 1G>T	Splice	Het	AR	rs762660468	P
			*OTOF*	NM_194248.2:c.5566C>T:p.Arg1856Trp	Missense	Het	AR	rs368155547	LP
69	F	1	*OTOF*	NM_194248.2:c.5203C>T:p.Arg1735Trp	Missense	Het	AR	rs1172714485	LP
			*OTOF*	NM_194248.2:c.2985C>A: p.Cys995Ter	Nonsense	Het	AR	Novel	P
70	M	0.17	*TIMM8A*	NM_004085.3:c.223C>T:p.Gln75*	Nonsense	Hemi	XLR	Novel	P
71	F	1.5	*OTOF*	NM_001287489.1:c.5815C>T:p.Arg1939Trp	Missense&splice	Hom	AR	rs368790049	P
74	M	1	*ACTG1*	NM_001199954.1:c.377C>T:p.Thr126Ile	Missense	Het (*de novo*)	AD	rs876657740	LP
75	M	0.5	*OTOF*	NM_194248.2:c.5108_5114 delinsTCTTCCTGGG: p.(Arg1703_Glu1705delinsLeuPheLeuGly)	Indel	Het	AR	PMID: 35982127	LP
			*OTOF*	NM_194248.2:c.709C>T:p.Arg237Ter	Nonsense	Het	AR	rs397515610	P
15	M	9	*ATP1A3*	NM_152296.4:c.2452G>A:p.Glu818Lys	Missense	Het	AD	rs587777771	P
22	M	5.5	*TWNK*	NM_021830.5:c.1172G>A:p.Arg391His	Missense	Het	AR	rs556445621	LP
			*TWNK*	NM_021830.5:c.1844G>C:p.Gly615Ala	Missense	Het	AR	rs764752550	LP
46	M	12	*AIFM1*	NM_004208.3:c.434C>T:p.Ala145Val	Missense	Hemi	XLD	rs724160015	LP
51	M	12	*AIFM1*	NM_004208.3:c.1773C>G:p.Ile591Met	Missense	Hemi	XLD	Novel	LP
68	M	10	*AIFM1*	NM_001130846.2:c.649A>G:p.Arg217Gly	Missense	Hemi	XLD	Novel	LP

Not determined: DNA samples of families were not available.

#### 3.6.1. OTOF

Of 13 patients caused by mutations in *OTOF* attributed to CI, CAP score (*n* = 10) improved dramatically from 0.10 ± 0.32 to 6.20 ± 1.32 (*p* < 0.001), and SIR score (*n* = 13) increased significantly from 1.00 ± 0.00 to 3.83 ± 1.10 (*p* < 0.001), while in proband 71, SIR score increased to 2 after the operation, which indicated the poor effect of CI.

#### 3.6.2. AIFM1

In three subjects (cases 46, 51, 68), simple hearing loss was identified to be caused by mutations in *AIFM1*. In case 46, the average hearing threshold improved from 78.25 dB to 40 dB, the CAP score increased from 0 to 3, and the SIR score improved from 1 to 5 after three years of operation. In case 51, the speech recognition score of monosyllables, disyllables, and sentences in quiet was 0, 0, and 0% before implantation. In comparison, the score was 82, 88, and 98%, respectively, after 1.5 years of implantation in the left ear. In patient 68, the CAP score increased from 2 to 5, and the SIR score increased from 1 to 3 after one year of implantation.

#### 3.6.3. TIMM8A

Two mutations in *TIMM8A* were detected in patients 61 and 70, respectively. Case 61 was found to have progressive hearing impairment at two years old, and he had no other obvious abnormalities until this report. After six months of implantation in the right ear, his average hearing threshold improved from 116.25 dB to 40 dB, his CAP score increased from 0 to 5, and his SIR score increased from 1 to 4. Case 70 was noticed to have hearing loss at two months old, with the molecular diagnosis being established at the age of one year. The maternal aunt of Proband 70 also had hearing loss with the same pathogenic variant as Proband, which was revealed when he was 30. Case 70 and his maternal aunt had no other obvious abnormalities. After one year of implantation in the left ear, the average hearing threshold of case 70 improved from 91.25 dB to 36.25 dB, the CAP score increased from 0 to 6, and the SIR score rose from 1 to 3.

#### 3.6.4. ATP1A3

For case 15, progressive hearing loss combined with tinnitus was noticed at nine years of age, and no other abnormality was reported. The mother of Proband was also affected by hearing loss combined with nystagmus and ataxia at three years old. A known pathogenic variant in *ATP1A3* (p.Glu818Lys) (Han et al., 2017) was found in Proband and confirmed to be inherited from his mother. The average hearing threshold for this patient improved from 71.25 dB to 35 dB. The speech recognition score of vowel, consonant, disyllable, and tone was 72, 80, 70, and 72%, respectively by the MESP test; the SIR score increased from 1 to 3 after unilateral cochlear implantation of half a year.

#### 3.6.5. TWNK

In case 22, compound heterozygote variants in the *TWNK* gene were detected. He walked unsteadily and fell quickly after three years old; both eyes had strabismus and astigmia when he was four years old and progressive hearing loss at half past five years old. He was later diagnosed as AN with profound hearing impairment. His speech, understanding, emotion management, and body development were delayed. The sibling of Proband had similar clinical manifestations and had the same compound heterozygote variants in *TWNK*. Accordingly, these two siblings were diagnosed with Perrault syndrome. For the Proband, after one year of implantation, the average hearing threshold improved from 98.75 dB to 38.25 dB, and vowel, consonant, disyllable, and tone recognition scores in the quiet field were 36, 36, 36, and 56%, respectively. In contrast, the speech recognition rate was 0% for all subtests before CI by the MESP test. The CAP score increased from 0 to 6, and the SIR score improved from 1 to 3 after two years of implantation.

Case 30 had congenital hearing loss combined with ovarian dysfunction, manifested as primary amenorrhea and undeveloped mammary gland. Compound heterozygote variants in the *TWNK* gene were also identified in this patient. The CAP score increased from 1 to 4, and the SIR score rose from 1 to 3 after two years of implantation.

#### 3.6.6. TWIST1

**Figure 2:**
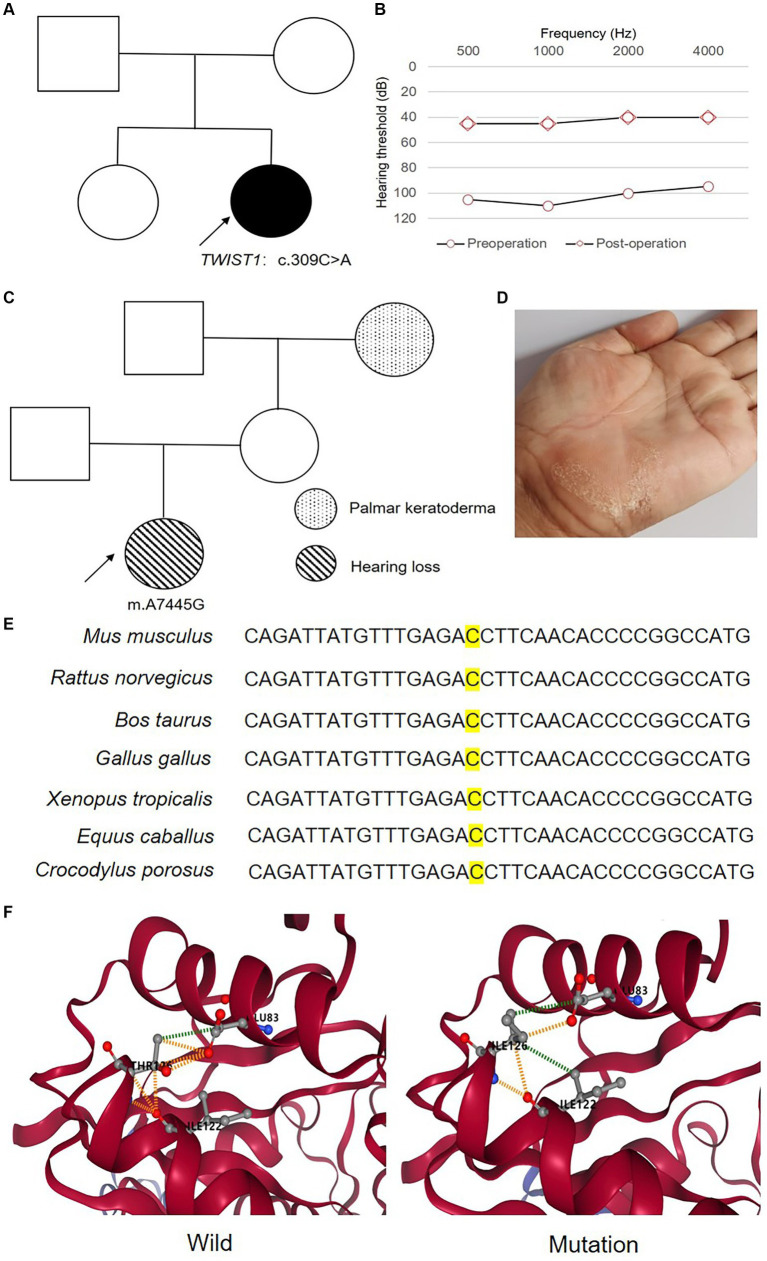
Case 11, 39, 74 caused by molecular etiologies related to AN. **(A)** Pedigree of case 11 caused by c.309C>A in *TWIST*. **(B)** Hearing thresholds of case 11 before and after CI. **(C)** Pedigree of case 39 caused by m.A7445G in *MT-CO1*. **(D)** Palmar keratoderma on the left hand of maternal grandmother of Proband. **(E)** A *de novo* variant identified in *ACTG1* in case 74 and the high conservation of the base in *ACTG1* among species. **(F)** The variant of p.T126I would impact the secondary bone between T126 and I122, E83, leading to the reduction of affinity between subdomains or amino acid chains (ΔΔG affinity = −0.136 kcal/mol).

Case 11 had congenital hearing loss and was later diagnosed as AN with profound hearing impairment. This patient had ptosis on the right eye, hypertelorism, skull shape asymmetry, and bilateral semicircular canal abnormalities. She had a pathogenic variant in the *TWIST1* gene (rs104894054). Accordingly, the patient was diagnosed as having Saethre-Chotzen syndrome. The average hearing threshold improved from 102.5 dB to 35 dB, the CAP score increased from 0 to 5, and the SIR score was raised from 1 to 2 after six months of implantation (Figure 2).

#### 3.6.7. m.A7445G

For case 39, profound hearing loss was detected at half years old and later diagnosed as AN. Genetic testing revealed m.A7445G in homoplasmic status, with no other variant related to hearing loss detected in this case. This variant was inherited from the mother, whose hearing was normal. The maternal grandmother of Proband had normal hearing but palmar keratoderma on the left hand (Figure 2). The m.A7445G variant was identified as the molecular cause of hearing loss of the Proband. After four years of implantation, the CAP score was raised from 0 to 7, and the SIR score increased from 1 to 5.

#### 3.6.8. ACTG1

Severe hearing loss was found at one year old in case 74, and no other obvious abnormalities were found till this report when this case was 13 years old. A likely pathogenic *de novo* variant in *ACTG1* was identified by Trio exome sequencing as the cause of this disease, and no other variant leading to hearing loss was revealed in this patient. After seven years of implantation, the average hearing threshold improved from 70.25 dB to 43.75 dB, the CAP score increased from 2 to 5, and the SIR score was raised from 1 to 2.

#### 3.6.9. The copy-number variant

Trio exome sequencing found a pathogenic *de novo* CNV to be a possible cause of AN in case 56 (hearing levels of this patient’s parents are normal). In addition, three variants with uncertain significance were also detected in this patient, including c.3041C>T in *MYO7A* (NM_000260.3), c.1007G>T in *USH1C* (NM_001297764.1), and c.1461G>T in *PDZD7* (NM_001195263.1), which was inherited from father, mother, mother of this patient, respectively. Profound hearing loss was observed when case 56 was one year old, and the parents of this case reported no other abnormalities until this report. After four years of implantation, the average hearing threshold improved from 97.75 dB to 30 dB after implantation of half years. The CAP score was increased from 0 to 7, and the SIR score was raised from 1 to 5.

### 3.7. Candidate impact factors on speech performances after cochlear implantation

**Figure 3:**
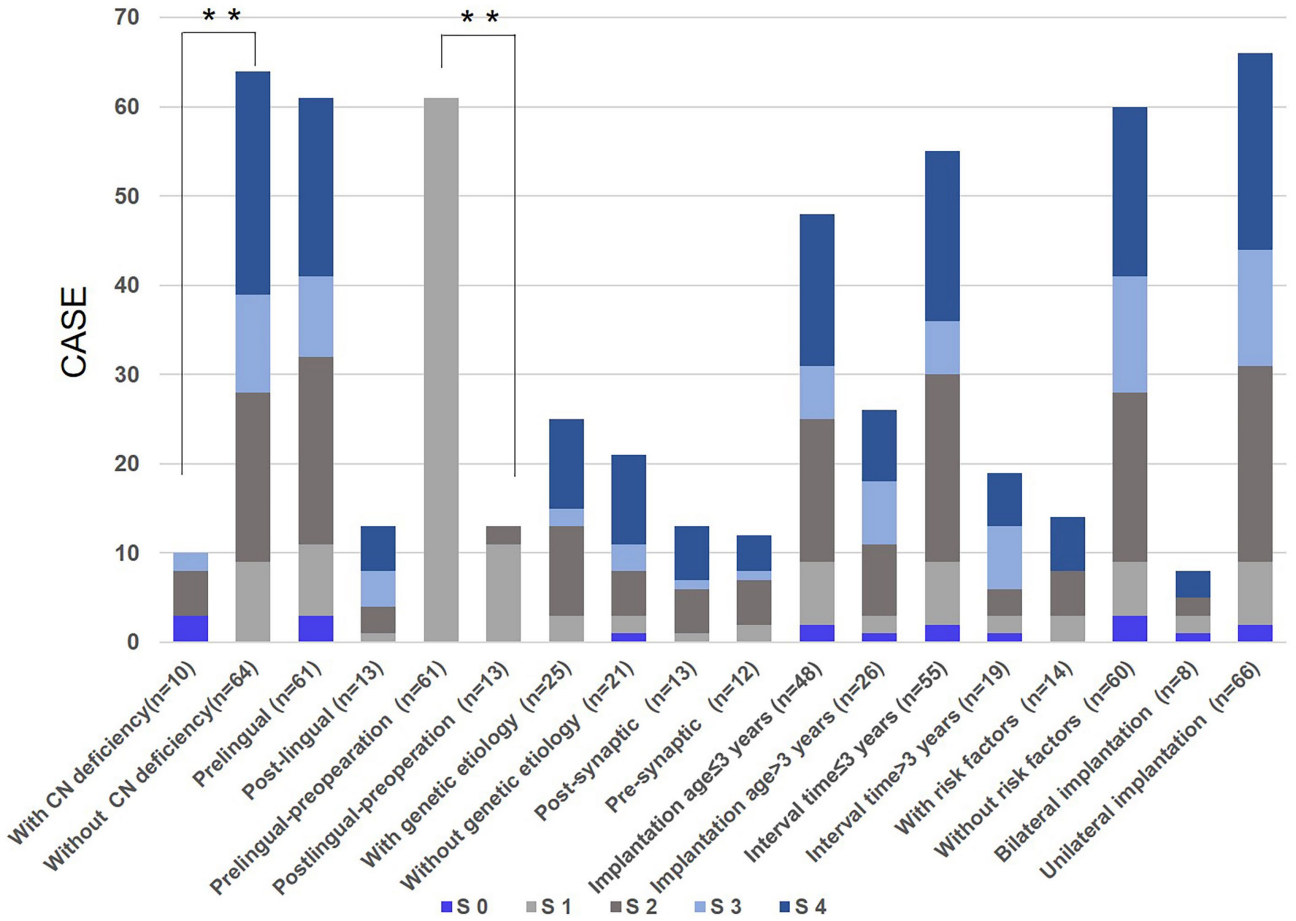
Candidate impact factors on speech outcomes due to cochlear implantation (CI). S 0: number of cases whose SIR score after CI did not improve compared with that before CI; S 1, S 2, S 3, S 4: number of cases in whom speech intelligibility rating (SIR) score after CI was improved by one, two, three, four compared with that before CI, respectively; Whitney U test was applied for significance analysis and statistical significance was defined as ^**^p < 0.01.

In this study, based on SIR score (*n* = 74) before and after CI, candidate impact factors on speech performances attributed to CI were analyzed, including the age of onset, age of implantation, with or without risk factors, with or without CN deficiency, bilateral or unilateral implantation, etc. (Figure 3). As we observed, no significant difference in the speech capabilities was observed between subjects with implantation age younger or older than 3 years of age (*p* = 0.671), subjects with an interval time between onset age and implantation age less or more than three years (*p* = 0.502), subjects with or without risk factors (*p* = 0.925), and subjects with bilateral or unilateral implantation (*p* = 0.394); while speech outcomes of patients with no CN deficiency were better than those of subjects with CN deficiency (*p* = 0.008). As for subjects with prelingual and post-lingual hearing loss, there was no significant difference in speech outcomes after CI (*p* = 0.260), but for speech performances before IC, patients with post-lingual onset were better than that of subjects with prelingual onset (*p* = 0.002).

Similarly, 46 patients participating in the genetic testing had no significant difference in speech outcomes between subjects with or without positive genetic etiology (*p* = 0.597). Furthermore, there was no significant difference in speech performance for subjects with *OTOF* variants and subjects with remaining genetic lesions (*p* = 0.503).

## 4. Discussion

### 4.1. Outcomes of cochlear implantation in patients with auditory neuropathy

Auditory and speech outcomes of CI in subjects with AN are always the primary issues to be addressed. Though many studies have focused on this topic (Alzhrani et al., 2019; Shearer and Hansen, 2019), it is still difficult for patients or their parents to decide on CI. In this retrospective study, 75 unrelated patients with AN (61 subjects with the prelingual onset and 14 with post-lingual onset) were enrolled to evaluate auditory and speech performances after CI. For both prelingual and post-lingual recipients, there was a significant improvement in hearing and speech performances compared with those at pre-operation, according to the results of hearing threshold, CAP score, and speech audiometry, SIR score. Generally, cochlear implantation is efficient for patients with AN with severe to profound hearing impairment or those who could not benefit sufficiently from hearing aids.

### 4.2. Risk factors

In the present study, CN deficiency was detected in 10 patients. Second, neonatal hyperbilirubinemia was detected in five patients, including two with other disorders. In another patient, bilirubin encephalopathy combined with neonatal RH hemolysis was also detected. This finding confirms the association between hyperbilirubinemia and AN occurrence (Almishaal et al., 2022). Of 10 patients with CN deficiency and 14 patients identified with other risk factors, nine underwent genetic testing, while molecular etiology was identified in none of these patients. In addition, none of the 75 patients underwent CMV screening. As a result, the risk factor of CMV infection was not analyzed here.

### 4.3. Impact factors

Speech performances of the patient subgroup with CN deficiency were significantly poorer than those of the subgroup with no CN deficiency after CI. At the same time, other candidate impact factors evaluated here had no impacts on CI outcomes, indicating that we should be cautious with the decision of CI in patients with CN deficiency. For 25 patients with a positive molecular diagnosis, there was no significant difference in speech outcomes of patients with mutations in *OTOF*, which was believed to be related to presynaptic pathology, and mutations from the remaining eight genes or CNV, that were thought to be associated with postsynaptic pathology or affecting spiral ganglion and auditory nerve (Shearer and Hansen, 2019; Chaudhry et al., 2020).

### 4.4. Genetic etiology

Saethre-Chotzen syndrome is a rare, dominant condition characterized by craniosynostosis and syndactyly (Pelc and Mikulewicz, 2018). Diagnosis of this syndrome is based on typical clinical manifestations and identification of pathogenic variants in the *TWIST1* gene. Sensorineural, conductive, and mixed hearing loss might be identified in this condition (Rosen et al., 2011). However, to our knowledge, hearing loss type of AN had never been reported in this syndrome before, which would now broaden the phenotype spectrum of this disorder.

A variant of m.A7445G in mitochondrial DNA was implicated in sensorineural hearing loss and nonepidermolytic palmoplantar keratoderma, with incomplete penetrance and variable clinical findings (Maász et al., 2008). The severity of hearing loss caused by m.A7445G varied from mild to profound, while onset age ranged from infancy to adulthood (Maász et al., 2008; Matsushima et al., 2018), although m.A7445G was first reported to be related to AN in this study.

*ACTG1* gene, encoding γ-actins, is the responsive gene of DFNA20/26 and Baraitser–Winter cerebrofrontofacial syndrome (Verloes et al., 2015; Sorrentino et al., 2021). The characteristic clinical phenotype of DFNA20/26 is progressive post-lingual hearing loss. However, congenital severe isolated hearing loss caused by a variant in *ACTG1* was also reported earlier (Lee et al., 2018). Here, case 74 had a likely pathogenic *de novo* variant in *ACTG1* (T126I) was identified. The wild type of this variant was highly conserved among species (Figure 2; Liu et al., 2008). Moreover, the secondary bond between T126 and I122 (present in subdomain 4) and E83 (present in subdomain 1) amino acid residues would change in mutation type, which would reduce the affinity between amino acid chains or subdomains of protein^1^ (Figure 2; Liu et al., 2008).

A pathogenic CNV encompassing 1.08 Mb, a recurrent microdeletion in 7p22.1 (Shimojima et al., 2016), was detected and identified as the possible genetic cause in case 56. This CNV covered 12 genes, including *SDK1*, *FOXK1*, *AP5Z1*, *RADIL*, *MMD2*, *RBAK*, *WIPI2*, *SLC29A4*, *TNRC18*, *FBXL18*, *ACTB*, and *RNF216*. Among these genes, like *ACTG1*, *ACTB* encoding β-actins (β- and γ-actin differ at their conserved N-terminal ends by only 4 amino acids) is also associated with Baraitser–Winter cerebrofrontofacial syndrome. Clinical presentations of this syndrome identified sensorineural hearing loss (Verloes et al., 2015). Additionally, haploinsufficiency of *ACTB* has been reported to be the possible responsive gene of 7p22.1 microdeletion (Palumbo et al., 2018). Therefore, we inferred that the heterozygous deletion of the *ACTB* gene might be the main cause of AN in this case.

Perrault syndrome characterizes sensorineural hearing loss with or without progressive neurological deficit in both sexes, combined with ovarian dysfunction only in females (Fiumara et al., 2004). *TWNK* is one of the responsive genes of this syndrome. Meanwhile, the molecular etiology of this syndrome was not identified in approximately 60% of the patients (Lerat et al., 2016; Demain et al., 2017). Herein, we present the first report of a Chinese patient with AN associated with Perrault syndrome, further confirming AN as an audiological feature of this syndrome (Ołdak et al., 2017; Forli et al., 2021). In case 58, hearing loss occurred at eight years of age, combined with primary amenorrhea and hypoplasia of the uterus, and the genetic cause could not be revealed by gene testing. This patient was also diagnosed with Perrault syndrome.

### 4.5. Presynaptic and postsynaptic mechanisms of AN

The presynaptic processes of AN encompass both dysfunction and/or loss of inner hair cells, as well as abnormalities in the ribbon synapses in these cells. The postsynaptic mechanisms of AN encompass various pathological conditions, including abnormalities in dendritic nerve terminals, axonal neuropathies, disorders affecting auditory ganglion cells, myelin disorders, hypoplasia of the auditory nerve, and auditory nerve conduction disorders (Rance and Starr, 2015). CI technology is specifically engineered to directly stimulate the auditory nerve, circumventing the signal transmission process between ribbon synapses in inner cells and terminal dendrites of the auditory nerve. The CI outcomes are influenced by the specific locations of malfunction (Shearer and Hansen, 2019). Oxygen deprivation-induced auditory system impairment was linked to inner hair cell dysfunction, and the auditory system’s sensitivity to hyperbilirubinemia involved presynaptic terminal deficits and damages in neuronal Ca^2+^ homeostasis, while congenital auditory nerve malformation was found in the postsynaptic site (Rance and Starr, 2015; Moser and Starr, 2016). The findings of this study indicate that the outcomes of CI in patients with CN deficiency were poorer than other patients.

Regarding genetic causes, the products of *OTOF* have been identified as playing a role in the exocytosis process of glutamatergic ribbon synapses. It has been reported that mutations in *OTOF* can lead to presynaptic synaptopathy (Moser and Starr, 2016). The study findings indicate that patients with *OTOF* mutations have demonstrated favorable outcomes of CI (Zheng and Liu, 2020). Mutations occurring in the *ATP1A3* gene have probably contributed to the development of postsynaptic synaptopathy (Han et al., 2017). The present study has demonstrated that individuals who have AN resulting from *ATP1A3* mutations may have potential advantages from CI (the SIR score ≥ 3, or the maximum speech recognition score > 60% after CI). Similarly, it has been reported that two of four CAPOS patients caused by the same mutation as we have identified in this study have markedly benefitted from CI (Tranebjærg et al., 2018). The study indicated a correlation between mutations in *TIMM8A* and the degeneration of cochlear, vestibular, and optic neurons (Bahmad et al., 2007). Additionally, patients with mutations in *AIFM1* experienced delayed development of cochlear nerve hypoplasia (Zong et al., 2015). *TIMM8A* and *AIFM1* mutation has been identified as potential factors contributing to the development of postsynaptic neuropathy (Shearer and Hansen, 2019). Nevertheless, as previously documented, three individuals exhibiting mutations in *AIFM1* and two patients with *TIMM8A* mutations all experienced positive outcomes from CI. The precise pathological mechanisms and specific locations of lesions associated with mutations in *TWNK*, *TWIST1*, m.A7445G, *ACTG1*, and the pathogenic CNV in 7p22.1, remain uncertain. CI outcomes are few and documented in individuals who possess mutations in the *TWNK* and *TWIST1* genes, as well as pathogenic CNVs in the 7p22.1 region. Positive outcomes have been obtained in patients with sensory hearing loss attributed to *m.A7445G* following CI (Love and Bird, 2013), same results have been observed in individual with AN (case 39) in this study as well. Previous reports have indicated that patients with mutations in *ACTG1* gene exhibited satisfactory speech performances after CI (Liu et al., 2019). However, in the present case (case 74), the outcomes of CI did not meet the anticipated level of speech recognition. Understanding the specific locations of lesions can provide valuable insights for guiding the therapeutic therapy of AN.

### 4.6. Limitation

A few limitations of this study include the relatively small subject size, especially leading to the small size of some subgroups in the analysis of impact factors on speech performances. The auditory and speech examinations administered to the recruited patients varied due to factors such as their age and hearing and speech abilities and experiences. In the interim, the length of CI among patients varied from six months to nine years, a factor that was considered significant in the context of speech rehabilitation.

## 5. Conclusion

In conclusion, 82.67% (62/75) of enrolled patients in the present study could benefit from CI (the SIR score of 3 to 5, or the maximum speech recognition score > 60% after CI). Ten patients were CN deficient whose speech performances after CI were poorer than those who were not CN deficient (*p* = 0.008). Other risk factors were identified in 14 patients, and the leading factor was neonatal hyperbilirubinemia. Molecular diagnosis was established in 25 (54.35%) of 46 patients participating in the comprehensive genetic testing.

## Data availability statement

The datasets presented in this study can be found in online repositories. The names of the repository/repositories and accession number(s) can be found in the article/Supplementary material.

## Ethics statement

The studies involving humans were approved by the Ethics Committee of Chinese PLA General Hospital. The studies were conducted in accordance with the local legislation and institutional requirements. Written informed consent for participation in this study was provided by the participants’ legal guardians/next of kin.

## Author contributions

JW: Formal analysis, Investigation, Writing – original draft. JC: Formal analysis, Investigation, Writing – original draft. ZD: Data curation, Writing – original draft. JF: Data curation, Writing – original draft. QW: Formal analysis, Writing – original draft. PD: Conceptualization, Writing – review & editing. DH: Conceptualization, Writing – review & editing.

## Footnotes

^1^ biosig.unimelb.edu.au/mcsm_ppi2/
