# Supplementary material for: Outcomes of cochlear implantation in 75 patients with auditory neuropathy
Source: Front Neurosci. 2023 Nov 13;17:1281884. doi: 10.3389/fnins.2023.1281884 (PMC10679445; doi:10.3389/fnins.2023.1281884)
Supplement: Supplementary file 1 [file Table_1.DOCX]

Supplementary Material

# Supplementary Table

Table S1. The information of enrolled patients in this study

| Patient | Gender | Onset/aware-ness age (years old) | Implanta-tion age | Age of auditory/speech tests | Affected ear | Implanta-tion ear | Inner ear/auditory nerve malformation | Risk factor | Family history | Molecular etiology | CAP  pre-CI post-CI | | SIR  pre-CI post-CI | | |
| --- | --- | --- | --- | --- | --- | --- | --- | --- | --- | --- | --- | --- | --- | --- | --- |
| 1 | F | 1 | 1.58 | 3.5 | B | L |  |  |  | Not found |  |  | 1 | 2 |  |
| 2 | M | 1 | 3 | 5 | B |  |  |  |  | Not found | 0 | 7 | 1 | 5 |  |
| 3 | M | 0 | 1.17 | 3.5 | B | R | bilateral absent CN |  |  | Not found | 0 | 5 | 1 | 3 |  |
| 4 | F | 1 | 1.25 | 3.75 | B | R | bilateral cochlear aperture hypoplastic, right internal auditory canal hypoplastic |  |  | Not found | 0 | 6 | 1 | 4 |  |
| 5 | M | 1.5 | 5 | 7 | B | B |  |  |  | Not found | 0 | 7 | 1 | 5 |  |
| 6 | M | 1 | 2 | 4 | B | L |  |  |  |  | 0 | 7 | 1 | 5 |  |
| 7 | M | 1 | 2 | 4 | B | R |  | expose to ototoxic medicine |  | Not found | 0 | 7 | 1 | 5 |  |
| 8 | M | 7 | 14 | 16 | B | L |  |  |  |  |  |  | 1 | 2 |  |
| 9 | M | 2 | 2 | 4 | B | R |  |  |  | Not found | 0 | 7 | 1 | 4 |  |
| 10 | F | 4 | 6.5 | 10.5 | B | L |  |  | Yes | Not found | 0 | 6 | 1 | 5 |  |
| 11 | F | 0 | 3.3 | 3.8 | B | R | bilateral semicircular canal abnormal |  |  | *TWIST1* | 0 | 5 | 1 | 2 |  |
| 12 | F | 3 | 3.5 | 5.5 | B | R |  | meningitis |  |  | 0 | 7 | 1 | 5 |  |
| 13 | M | 2 | 13.5 | 15.5 | B | R | enlarged vestibular aqueduct |  |  |  |  |  | 1 | 4 |  |
| 14 | M | 0.25 | 1 | 3 | B | R |  | neonatal septicemia combined with intrauterine distress and mixed acidosis |  | Not found | 0 | 7 | 1 | 5 |  |
| 15 | M | 9 | 11 | 11.5 | B | R |  |  | Yes | *ATP1A3* |  |  | 1 | 3 |  |
| 16 | F | 1 | 1.5 | 3.5 | B | R |  |  |  |  | 1 | 7 | 1 | 5 |  |
| 17 | F | 2 | 2.33 | 4.33 | B | L | bilateral cochlear malformation |  |  |  | 0 | 6 | 1 | 4 |  |
| 18 | M | 1.17 | 1.25 | 3.25 | B | L |  |  |  |  | 4 | 7 | 1 | 4 |  |
| 19 | F | 10 | 22 | 24 | B | R |  |  |  |  | 0 | 7 | 1 | 5 |  |
| 20 | M | 1 | 5.17 | 8.17 | B | B |  |  |  | *OTOF* |  |  | 1 | 5 |  |
| 21 | F | 2 | 2.17 | 4.17 | B | B |  |  |  | *OTOF* | 0 | 7 | 1 | 5 |  |
| 22 | M | 5.5 | 6.5 | 8.5 | B | R |  |  | Yes | *TWNK* | 0 | 6 | 1 | 3 |  |
| 23 | F | 1.58 | 1.58 | 5.58 | L | B | right internal auditory canal hypoplastic |  |  |  | 0 | 3 | 1 | 2 |  |
| 24 | M | 2 | 2.33 | 3.33 | B | L | bilateral hypoplastic CN |  |  |  | 0 | 5 | 1 | 3 |  |
| 25 | M | 1.5 | 1.5 | 3.5 | B | R |  | neonatal RH hemolysis combined with acute bilirubin encephalopathy |  |  | 2 | 7 | 1 | 5 |  |
| 26 | F | 1 | 1.75 | 3.75 | B | R |  |  |  | Not found | 0 | 3 | 1 | 3 |  |
| 27 | F | 0 | 2.5 | 4.5 | B | R | bilateral internal auditory canal hypoplastic |  |  |  | 0 | 4 | 1 | 2 |  |
| 28 | M | 1 | 2.17 | 4.17 | B | R |  |  |  |  | 0 | 5 | 1 | 3 |  |
| 29 | F | 25 | 32 | 34 | B | L |  |  |  |  | 0 | 7 | 2 | 5 |  |
| 30 | F | 0 | 7.5 | 9.5 | B | R |  |  |  | *TWNK* | 1 | 4 | 1 | 3 |  |
| 31 | F | 0.83 | 2.25 | 4.25 | B | R |  |  |  | *OTOF* | 0 | 6 | 1 | 3 |  |
| 32 | M | 2 | 8.5 | 10.5 | B | R |  | hypoxic-ischemic encephalopathy |  | Not found | 0 | 5 | 1 | 3 |  |
| 33 | M | 1 | 1 | 3 | B | R |  |  |  |  | 0 | 5 | 1 | 3 |  |
| 34 | M | 0 | 2.5 | 4.5 | B | R |  |  |  | *OTOF* |  |  | 1 | 3 |  |
| 35 | M | 1 | 2.75 | 4.75 | B | R |  |  |  |  | 1 | 4 | 1 | 3 |  |
| 36 | M | 1 | 3 | 4 | B | R |  |  |  | Not found | 0 | 6 | 1 | 5 |  |
| 37 | F | 0 | 1.33 | 3.33 | B | R |  |  |  | *OTOF* | 0 | 7 | 1 | 5 |  |
| 38 | F | 1.5 | 2 | 4 | B | L | bilateral hypoplastic CN, bilateral vestibule and semicircular canal abnormality |  |  |  | 1 | 7 | 1 | 3 |  |
| 39 | F | 0.5 | 2.67 | 4 | B | L |  |  | Yes | A7445G | 0 | 7 | 1 | 5 |  |
| 40 | F | 0.5 | 2.5 | 4.5 | B | R |  |  |  | *OTOF* | 0 | 7 | 1 | 5 |  |
| 41 | F | 1 | 1.58 | 3.58 | B | R | bilateral cochlear and vestibule abnormal, right internal auditory canal hypoplastic |  |  |  | 0 | 7 | 1 | 4 |  |
| 42 | F | 0.5 | 0.75 | 2.75 | B | R | bilateral hypoplastic CN |  |  |  | 0 | 5 | 1 | 3 |  |
| 43 | F | 16 | 29 | 29.5 | B | L | bilateral hypoplastic CN |  |  |  |  |  | 1 | 4 |  |
| 44 | M | 2.25 | 2.5 | 4.5 | B | R |  | premature |  | Not found | 0 | 7 | 1 | 5 |  |
| 45 | F | 12 | 28 | 30.5 | B | R | bilateral hypoplastic CN |  |  |  |  |  | 1 | 4 |  |
| 46 | M | 12 | 21 | 24 | B | L |  |  |  | *AIFM1* | 0 | 3 | 1 | 5 |  |
| 47 | M | 1 | 1.17 | 3.17 | B | L |  |  |  | *OTOF* | 0 | 7 | 1 | 3 |  |
| 48 | M | 0.67 | 3.25 | 5.25 | B | L |  | trauma |  |  | 0 | 7 | 1 | 3 |  |
| 49 | M | 1.08 | 1.75 | 3.75 | B | L |  | development delay |  | Not found | 0 | 4 | 1 | 3 |  |
| 51 | M | 12 | 34 | 35.5 | B | L |  |  | Yes | *AIFM1* |  |  | 1 | 5 |  |
| 52 | F | 0.91 | 0.91 | 2.91 | B | R |  |  |  | Not found | 1 | 7 | 1 | 5 |  |
| 53 | F | 0.75 | 0.75 | 2.75 | B | L |  |  |  | Not found | 2 | 6 | 1 | 5 |  |
| 54 | M | 8 | 16 | 18 | B | R |  |  |  |  |  |  |  |  |  |
| 55 | F | 0 | 0.75 | 2.75 | B | R |  |  |  | *OTOF* | 0 | 7 | 1 | 5 |  |
| 56 | F | 1 | 2.96 | 6.96 | B | R |  |  |  | CNV | 0 | 7 | 1 | 5 |  |
| 57 | F | 1 | 4.17 | 6.17 | R | B | bilateral hypoplastic CN, hypoplastic vestibular nerve (right) |  |  |  | 0 | 1 | 1 | 1 |  |
| 58 | F | 8 | 23 | 25 | B | L |  |  |  | Not found | 0 | 7 | 1 | 5 |  |
| 59 | F | 2 | 2.25 | 4.25 | B | R |  | neonatal hyperbilirubinemia |  |  | 0 | 6 | 1 | 3 |  |
| 60 | M | 13 | 20 | 22 | B | L |  |  |  |  | 2 | 7 | 2 | 5 |  |
| 61 | M | 2 | 8 | 8.5 | B | R |  |  |  | *TIMM8A* | 0 | 5 | 1 | 4 |  |
| 62 | F | 1 | 3 | 5 | B | L |  |  |  | *OTOF* | 0 | 5 | 1 | 3 |  |
| 63 | M | 2 | 4.17 | 6.17 | B | B |  | hydrocephalus |  |  | 0 | 3 | 1 | 3 |  |
| 64 | F | 2 | 2 | 4 | B | R |  |  |  | *OTOF* | 1 | 7 | 1 | 4 |  |
| 65 | F | 1 | 1 | 3 | B | L | bilateral hypoplastic CN, narrow internal auditory canal (right) |  |  | Not found | 0 | 2 | 1 | 1 |  |
| 66 | M | 2 | 2 | 4 | B | R |  |  |  | Not found | 0 | 5 | 1 | 2 |  |
| 67 | M | 1 | 1.46 | 3.46 | B | B |  | neonatal hyperbilirubinemia combined with favism |  |  | 0 | 2 | 1 | 2 |  |
| 68 | M | 10 | 21 | 22 | B | R |  |  |  | *AIFM1* | 2 | 5 | 1 | 3 |  |
| 69 | F | 1 | 2.13 | 4.13 | B | R |  |  |  | *OTOF* | 0 | 6 | 1 | 3 |  |
| 70 | M | 0.17 | 1.25 | 2.25 | B | L |  |  | Yes | *TIMM8A* | 0 | 6 | 1 | 3 |  |
| 71 | F | 1.5 | 2 | 4 | B | R |  |  |  | *OTOF* | 0 | 3 | 1 | 2 |  |
| 72 | F | 2 | 3.33 | 5.33 | L | B | bilateral hypoplastic CN |  |  | Not found | 0 | 3 | 1 | 3 |  |
| 73 | M | 0.58 | 0.92 | 2.92 | B | L | bilateral hypoplastic CN, bilateral cochlear aperture hypoplastic |  |  |  | 0 | 3 | 1 | 1 |  |
| 74 | M | 1 | 2.25 | 9.25 | B | R |  |  |  | *ACTG1* | 2 | 5 | 1 | 2 |  |
| 75 | M | 0.5 | 1.58 | 3.58 | B | R |  |  |  | *OTOF* |  |  | 1 | 5 |  |
| 76 | M | 2 | 5.5 | 7.5 | B | L |  |  |  | Not found | 0 | 6 | 1 | 4 |  |

B: bilateral; L: left; R: right; CN: cochlear nerve
